# Tdap Vaccination Coverage During Pregnancy — Selected Sites, United States, 2006–2015

**DOI:** 10.15585/mmwr.mm6641a3

**Published:** 2017-10-20

**Authors:** Stephen Kerr, Carla M. Van Bennekom, Jennifer L. Liang, Allen A. Mitchell

**Affiliations:** ^1^Slone Epidemiology Center at Boston University, Boston, Massachusetts; ^2^Vaccines and Medications in Pregnancy Surveillance System; ^3^Meningitis and Vaccine Preventable Diseases Branch, Division of Bacterial Diseases, CDC.

Tetanus toxoid, reduced diphtheria toxoid, and acellular pertussis (Tdap) vaccine is recommended during the third trimester of each pregnancy to provide protection to newborns, who are at risk for pertussis-related morbidity and mortality (*1*). As part of its case-control surveillance study of medications and birth defects, the Birth Defects Study of the Slone Epidemiology Center at Boston University (the Birth Defects Study) has recorded data on vaccinations received during pregnancy since 2006. Among 5,606 mothers of infants without structural birth defects in this population (control group), <1% had received Tdap vaccine before 2009. By 2012, the percentage of mothers of infants in the control group (control infants) who had received Tdap increased to approximately 9%, and then in 2013 and continuing through 2015, increased markedly, to 28% and 54%, respectively. As the prevalence of maternal Tdap vaccination increased, so did the proportion of pregnant women who received Tdap in the third trimester, as recommended (94%–100% from 2010 to 2015). The vast majority of Tdap vaccinations (96%) were received in a traditional health care setting (e.g., the office of the woman’s obstetrician or primary care physician or her prenatal clinic). Increasing vaccination coverage during pregnancy could help reduce the impact of pertussis on infant morbidity and mortality.

Pertussis is a highly contagious disease, but mortality is highest among newborns: almost all pertussis-associated deaths occur within the first 2 months of life (*2*), when these infants are too young to receive primary pertussis vaccinations. To provide infants with indirect protection from pertussis, in 2006, the Advisory Committee on Immunization Practices (ACIP) recommended postpartum Tdap administration to mothers, but noted that the vaccine could be administered during pregnancy.* In June 2011, ACIP changed the preferred timing of Tdap administration to mothers, recommending that previously unvaccinated pregnant women should receive Tdap after 20 weeks’ gestation (*3*). In October 2012, this recommendation was expanded to include all pregnant women during every pregnancy, with the optimal time for vaccination in the third trimester (*1*). A recent analysis reported 42% coverage with Tdap among pregnant women in 2013 (*4*). To assess the impact of the ACIP recommendations, trends in Tdap coverage in pregnancy were examined, along with the settings in which women received their vaccinations, from 2006 through 2015, using data from the Birth Defects Study.

During 1976–2015, the Birth Defects Study conducted surveillance using a case-control methodology described previously (*5*). Infants with major structural birth defects (case infants) were identified at study centers that, for the present analysis, included participating hospitals in the areas surrounding Boston, Massachusetts, Philadelphia, Pennsylvania, and San Diego, California, as well as statewide birth defects registries in New York and Massachusetts. Control infants were randomly selected each month from study hospitals’ discharge lists or statewide vital statistics records. Within 6 months of delivery, mothers of case and control infants were invited to participate in a computer-assisted telephone interview conducted by trained study nurses. Data were collected on demographic characteristics, lifestyle factors, reproductive history, illnesses, and medications used from 2 months before the last menstrual period through the end of pregnancy. Medication data included prescription and over-the-counter drugs, and for pregnancies that began in 2005 or later, any vaccines received during pregnancy. Women were asked to provide an exact date of vaccination, or if the vaccination date was not available, a range of possible dates, along with the setting or facility where the vaccine was administered (e.g., doctor’s office/prenatal clinic, workplace, school, pharmacy/supermarket, or government site). All women who reported receiving a vaccine were asked to provide a release allowing study personnel to contact the vaccine provider to validate the vaccine report. If vaccine records were not available, the maternal report was accepted (*6*). All vaccine doses received from the provider were recorded, and during the process of validating vaccination reports, study personnel sometimes discovered unreported Tdap vaccinations. For instance, a maternal report of influenza vaccination might lead to the recording of an unreported Tdap vaccination during pregnancy from the same provider.

This analysis of Tdap vaccination coverage was limited to pregnancies in women who gave birth to control infants during 2006–2015. Among women who reported receiving Tdap vaccine during pregnancy, the exact date of vaccination obtained from the vaccination record was used if the record was available; otherwise, the vaccination date the woman provided or the midpoint of the reported date range was used. Unvaccinated women were defined as those without provider-documented exposure who did not report receiving the Tdap vaccine, or whose reported Tdap vaccination took place before pregnancy or after delivery.

Among the 5,606 pregnant women who participated in the study during the 10 years included in the analysis, 849 (15%) received doses of Tdap during pregnancy. Among these doses, 83% were validated by provider records; the remaining 17% were ascertained only by maternal self-report. Fifty-nine (7%) Tdap doses were not reported by the mother and were identified during validation of other reported vaccinations (primarily influenza vaccine).

Tdap vaccination during pregnancy increased over the years of the study (Figure). Tdap vaccination during pregnancy occurred in <1% of women who delivered before 2010, but began to increase from 2010 (5%) to 2012 (9%); during 2010–2011, 83% of Tdap vaccinations documented in the study were received among mothers of control infants reported to the San Diego study center. Overall Tdap vaccination coverage approximately tripled in 2013 to 28%, with highest rates reported for mothers of control infants in Boston (34%) and Philadelphia (32%); reported vaccination rates continued to increase in 2014 (48%) and 2015 (54%) (2015 rates were 64% in Philadelphia, 56% in Boston, 52% in San Diego, and 44% in New York). Among all mothers giving birth, Tdap vaccination during the first trimester remained at approximately 1%, and vaccination in the second trimester ranged from 1% to 5%. Tdap vaccination in the third trimester increased from 4% in 2010 to 49% in 2015.

**FIGURE:**
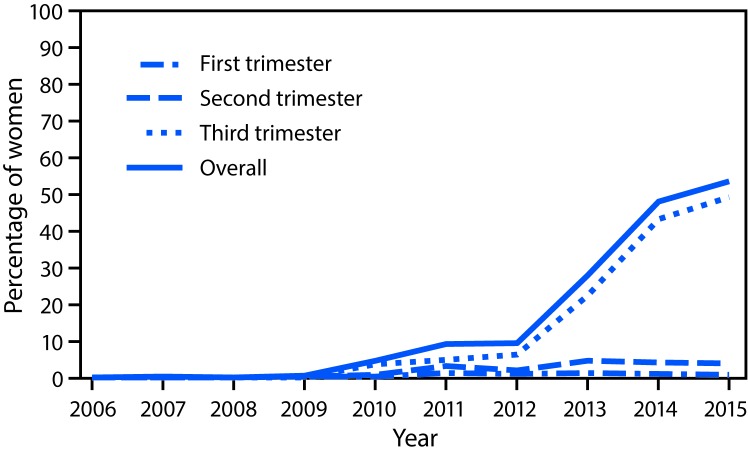
Percentage of women receiving tetanus toxoid, reduced diphtheria toxoid, and acellular pertussis (Tdap) vaccination during pregnancy, by trimester — selected sites,* United States, 2006–2015 * Birth Defects Study, Slone Epidemiology Center, Boston University. Study sites included participating hospitals in the areas surrounding Boston, Massachusetts, Philadelphia, Pennsylvania, and San Diego, California, as well as birth defects registries in New York and Massachusetts. Women included in the analysis were mothers of control infants (infants born without a structural birth defect).

Overall, 96% of Tdap vaccine doses received by pregnant women were administered in a traditional health care setting (e.g., the office of their obstetrician or primary care physician or their prenatal clinic). Among 4% of vaccine doses reported to have been administered in non-traditional health care settings, half were received at work or school settings, and one quarter each at pharmacy/supermarket or government settings. During the 10 years included in this analysis, the proportion of vaccine doses received by pregnant women in these settings remained stable; during 2010–2015, Tdap vaccinations administered in traditional health care settings accounted for 94%–100% of Tdap vaccine doses administered to pregnant women.

## Discussion

From 2006 to 2015, Tdap vaccination coverage among pregnant women in the Birth Defects Study who gave birth to control infants increased from <1% of births in 2009 to 9% in 2012, before increasing to 28% in 2013 and to >50% of births in 2015. These increases reflect the implementation of evolving ACIP recommendations, which currently recommend that all pregnant women be vaccinated during each pregnancy, ideally in the third trimester. Of note, before 2012, the San Diego study center accounted for the large majority of pregnant women in the Birth Defects Study who received Tdap; this was likely a result of California’s 2010 recommendation that all women of childbearing age be vaccinated with Tdap “preferably before pregnancy, but otherwise during or after pregnancy” in response to a statewide pertussis epidemic.^†^ The higher Tdap coverage beginning in 2010 has also been reported in two studies using the Vaccine Safety Datalink (*4*,*7*), and was likewise attributed to the 2010 recommendation in California (*7*). Tdap coverage in non-California sites remained low until 2012 (*7*). In both the Vaccine Safety Datalink and Birth Defects Study, marked increases were observed for all sites beginning in 2013; the Birth Defects Study data in this report indicate that this increase continued in 2014 and 2015. By 2015, all four Birth Defects Study centers (Boston, Philadelphia, San Diego, and New York) reported Tdap administration prevalences ranging from 44% to 64% among mothers of control infants.

The findings in this report are subject to at least three limitations. First, Tdap vaccination histories were ascertained by self-report and could be subject to misclassification; however, maternal reports of influenza vaccination in the Birth Defects Study were previously found to be accurate within a given trimester for 83% of women in this group (*8*), and 83% of Tdap vaccine doses reported in the current study were confirmed by vaccine providers’ records. Second, because the data was limited to reporting centers in only four U.S. locations, the study population might not be representative of the entire U.S. population. Finally, the small number of mothers vaccinated each year might have affected year-to-year variability.

Tdap vaccination during pregnancy increased substantially among mothers of control infants in the Birth Defects Study over the 10 years included in this report. Although approximately half of mothers who gave birth to control infants in the most recent year of the study received Tdap during pregnancy, this proportion remains far below the ACIP recommendation that all pregnant women be vaccinated during each pregnancy. Newborns at highest risk for pertussis-associated complications are too young to be vaccinated, but Tdap vaccination during pregnancy can reduce the potential for morbidity (*9*) and mortality in this vulnerable population. A recent U.S. study found that Tdap vaccination during the third trimester of pregnancy was 85% more effective than postpartum vaccination at preventing pertussis in infants aged <2 months (*10*). To help increase coverage of Tdap vaccination among pregnant women, resources are available for prenatal care providers and pregnant women at https://www.cdc.gov/pertussis/pregnant.

**Summary**

**What is already known about this topic?**

Infants are at risk for pertussis-related morbidity and mortality especially in the early months of life when they are too young to be vaccinated. Beginning in 2012, tetanus toxoid, reduced diphtheria toxoid, and acellular pertussis (Tdap) vaccine has been recommended for pregnant women during the third trimester of each pregnancy to provide protection to the newborn. A recent report indicated Tdap vaccination coverage during pregnancy was approximately 42% in women giving birth during 2013.

**What is added by this report?**

Among mothers of control infants participating in the Birth Defects Study of the Slone Epidemiology Center, which included pregnant women in New York and Massachusetts and the areas surrounding Philadelphia, Pennsylvania, and San Diego, California, Tdap vaccination coverage increased from <1% before 2010 to 28% in 2013, and reached 53% in 2015. Overall, 96% of Tdap vaccinations received by pregnant women in this study were administered in physicians’ offices or clinics.

**What are the implications for public health practice?**

Although Tdap vaccination coverage has increased in recent years and approximately half of pregnant women in this study who had a live birth in 2015 received Tdap, coverage for pregnant women remains far below the recommendation that every woman be vaccinated during each pregnancy. Increasing vaccination coverage during pregnancy could help reduce the impact of pertussis on infant morbidity and mortality.

## Footnotes

* https://www.cdc.gov/mmwr/preview/mmwrhtml/mm5540a10.htm.

† https://www.lahc.edu/includes/Pertussis%20Vaccine%20Info.pdf.
